# Comment on “Updated Meta‐Analysis of Left Bundle Branch Area Pacing Versus Right Ventricular Pacing in Conduction System Disorders: Insights From New Evidence”

**DOI:** 10.1002/clc.70346

**Published:** 2026-05-13

**Authors:** Taha Ibrahim, Muhammad Burhan, Syed Rayyan Ahmed, Ibrahim Nagmeldin Hassan

**Affiliations:** ^1^ Department of Medicine Dow University of Health Sciences Karachi Pakistan; ^2^ Department of Medicine University of Khartoum Khartoum Sudan

Dear Editor,

We read with eminent interest the updated evidence synthesis regarding the comparative effectiveness of Left Bundle Branch Pacing (LBBP) Versus Right Ventricular Pacing (RVP) in conduction system disorders by Ishaque et al. [1]. While the study provides an extensive synthesis of contemporary evidence, several methodological concerns warrant clarification.

First, the meta‐analysis pools randomized controlled trials and predominantly observational studies without stratified or sensitivity analyses based on study design. Given the inherent susceptibility of observational data to confounding and selection bias, this approach may inflate effect estimates, particularly for clinical outcomes such as mortality and heart failure hospitalization, thereby limiting causal inference. Furthermore, there is no subgroup introduction based on, for example, Study Design, follow‐up duration, or conduction system disorders despite encountering high heterogeneity among studies for the key evidence‐based outcomes. Besides all of this, there is no explicit substantial discussion about the causes of heterogeneity.

Second, there is substantial variability in follow‐up durations across included studies, ranging from short‐term (≤ 7 days) to several years. The authors report pooling of clinical outcomes irrespective of follow‐up timepoints, which raises concerns regarding the validity of time‐dependent endpoints such as mortality and hospitalization. Without time‐standardized analyses, pooled risk estimates may be misleading.

Third, the authors position their study as an updated meta‐analysis; there is insufficient transparency regarding continuity with prior evidence synthesis. The referenced earlier meta‐analysis included 11 studies [2], yet the current analysis includes 23 studies from previous studies without clearly discussing it. Furthermore, discrepancies between the number of studies presented in the PRISMA flow diagram and those contributing to individual forest plots are not adequately explained. Moreover, Leventopoulos et al. compared LBBP with biventricular pacing (BiVP), which represents a fundamentally different intervention from RVP. These modalities differ substantially in mechanism, clinical indication, and target population; therefore, they cannot be considered equivalent comparators [3, 4]. Any attempt to conceptually align or synthesize evidence across these distinct interventions risks violating the core PICO framework for the study by Ishaque et al. [1], thereby compromising the validity and interpretability of the comparative analysis.

Fourth, in evaluating changes in LVEF from baseline, Ishaque et al. [1] state that “the heterogeneity analysis corroborated the robustness of LVEF enhancement with LBBAP.” However, this interpretation is difficult to justify given the presence of substantial heterogeneity in the pooled analysis. High heterogeneity typically reflects significant between‐study variability and undermines confidence in the stability and generalizability of the effect estimate. A similar interpretative approach is observed in the analysis of LVEDD, where considerable heterogeneity is again downplayed while conclusions of robustness are maintained. Such inconsistencies between statistical findings and their interpretation raise concerns regarding the validity of the evidence synthesis and suggest a risk of overestimating the strength and reliability of the reported outcomes.

Taken together, while this meta‐analysis contributes valuable updated data, the methodological limitations outlined above suggest that the reported clinical benefits of LBBAP should be interpreted with caution. Future studies incorporating patient‐level data, standardized follow‐up durations, and robust stratified analyses are needed to more definitively establish the comparative effectiveness of LBBAP.

## Author Contributions

**Taha Ibrahim:** writing – original draft, writing – review and editing, literature review. **Muhammad Burhan:** writing – original draft, writing–review and editing, literature review, critical interpretation. **Syed Rayyan Ahmed:** writing – original draft, writing – review and editing, methodological input, critical interpretation. **Ibrahim Nagmeldin Hassan:** conceptualization, supervision, writing – review and editing, project administration. All authors read and approved this manuscript.

## Funding

The authors have nothing to report.

## Ethics Statement

The authors have nothing to report.

## Conflicts of Interest

The authors declare no conflicts of interest.

## Data Availability

The data that support the findings of this study are available from the corresponding author upon reasonable request.
